# Transcutaneous auricular vagus nerve stimulation attenuates inflammatory bowel disease in children: a proof-of-concept clinical trial

**DOI:** 10.1186/s42234-023-00124-3

**Published:** 2023-10-18

**Authors:** Benjamin Sahn, Kristine Pascuma, Nina Kohn, Kevin J. Tracey, James F. Markowitz

**Affiliations:** 1 Division of Pediatric Gastroenterology, Liver Diseases, & Nutrition, Steven & Alexandra Cohen Children’s Medical Center, Northwell Health, 1991 Marcus Ave, Suite M100, New Hyde Park, NY 11042 USA; 2 Feinstein Institutes for Medical Research, Manhasset, NY USA; 3 Biostatistics Unit, Office of Academic Affairs, New Hyde Park, NY USA

**Keywords:** Vagus nerve stimulation, Auricular, Inflammatory bowel disease, Crohn’s disease, Ulcerative colitis

## Abstract

**Background:**

Vagus nerve stimulation is an investigational anti-inflammatory therapy targeting the nervous system to modulate immune activity. This study evaluated the efficacy and safety of transcutaneous auricular VNS (ta-VNS) in patients with pediatric-onset Crohn’s disease (CD) or ulcerative colitis (UC).

**Methods:**

Participants were 10–21 years of age with mild/moderate CD or UC and fecal calprotectin (FC) > 200 ug/g within 4 weeks of study entry. Subjects were randomized to receive either ta-VNS targeting the cymba conchae of the external left ear, or sham stimulation, of 5 min duration once daily for a 2-week period, followed by a cross over to the alternative stimulation for an additional 2 weeks. At week 4, all subjects received ta-VNS of 5 min duration twice daily until week 16. Primary study endpoints were clinical remission, and a ≥ 50% reduction in FC level from baseline to week 16. Heart rate variability measurements and patient-reported outcome questionnaires were completed during interval and week 16 assessments.

**Results:**

Twenty-two subjects were enrolled and analyzed (10 CD, 12 UC). Six of 10 with CD had a wPCDAI > 12.5 and 6/12 with UC had a PUCAI > 10 at baseline, correlating to mild to moderate symptom activity. Among the 12 subjects with active symptomatic disease indices at baseline, clinical remission was achieved in 3/6 (50%) with CD and 2/6 (33%) with UC at week 16. Despite all subjects having FC levels ≥ 200 within 4 weeks of enrollment, five subjects (4 UC, 1 CD) had FC levels < 200 at the baseline visit and were excluded from the FC analysis. Of the remaining 17, median baseline FC was 907 µg/g (IQR 411–2,120). At week 16, 11/17 (64.7%) of those with baseline FC ≥ 200 had a ≥ 50% reduction in FC (95% CI 38.3—85.8). In the UC subjects, there was an 81% median reduction in FC vs baseline (833 µg/g; *p* = 0.03) while in the CD subjects, median reduction in FC at 16 weeks was 51% (357 µg/g; *p* = 0.09). There were no safety concerns.

**Conclusion:**

Noninvasive ta-VNS attenuated signs and symptoms in a pediatric cohort with mild to moderate inflammatory bowel disease.

**Trial Registration:**

NCT03863704—Date of registration 3/4/2019.

## Background

Chronic inflammatory bowel disease (IBD) comprising Crohn disease (CD) and ulcerative colitis (UC), is a global disease with rising prevalence, associated with significant health morbidity and very high health care costs substantially driven by therapeutics (Windsor and Kaplan 2019; Ng et al. 2017; Zhao et al. 2021; Peery et al. 2018). While many with IBD have benefited greatly from modern era biologic and small molecule therapies, unresolved chronic inflammation is common despite treatment, and is fundamental to the evolution of disease complications (Baumgart and Berre 2021). Therefore, a non-pharmacologic, non-toxic, lower cost anti-inflammatory therapy is necessary.

The inflammatory reflex is a vagus nerve mediated physiologic process by which the nervous system can attenuate peripheral inflammation (Rosas-Ballina et al. 2011). Proinflammatory cytokines and other molecules are detected by sensory neurons communicating to the central nervous system, leading to a counterregulatory response through the efferent vagus nerve and other neuronal pathways (Pavlov et al. 2018). Since the initial description of this cholinergic anti-inflammatory pathway by Tracey and colleagues in the early 2000s, (Borovikova et al. 2000; Tracey 2002) hundreds of studies have confirmed the critical influence of autonomic and enteric neural signaling pathways, such as the inflammatory reflex, on intestinal immune function, inflammation, epithelial barrier integrity, and T-regulatory cells in the gut (Brinkman et al. 2019; Meroni et al. 2019; Matteoli et al. 2014; Bai et al. 2007; Ghia et al. 2007, 2006; Teratani et al. 2020; Jin et al. 2017; Meregnani et al. 2011; Meroni et al. 2021). The field of bioelectronic medicine has capitalized on these discoveries toward using device technology to treat human inflammatory disease. Initial human studies engaging the inflammatory reflex through a surgically implanted vagus nerve stimulator have shown efficacy in treating rheumatoid arthritis (RA) (Koopman et al. 2016) and CD (Bonaz et al. 2016). In adults with CD, vagus nerve stimulation led to improvement in symptoms, inflammatory biomarkers, and endoscopic healing at 12 months of follow up (Sinniger et al. 2020). Until now, there have been no reports of successfully treating IBD in humans with non-invasive vagus nerve stimulation methods.

Transcutaneous auricular vagus nerve stimulation (taVNS) has been identified as a feasible method to elicit the inflammatory reflex in a noninvasive manner without requirement for an implantable device (Addorisio et al. 2019; Aranow et al. 2021; Hong et al. 2019). The auricular branch of the vagus nerve is a sensory nerve to the external ear including the cymba concha. Stimulation of this auricular nerve branch delivers afferent neuronal impulses, whereas cervically implanted devices deliver efferent and afferent nerve stimulation. Functional MRI studies have mapped stimulation of the auricular branch of the vagus nerve to activate the same brain regions as implanted devices, confirming this nerve as a suitable noninvasive target to administer vagus nerve stimulation (Frangos et al. 2015). Use of taVNS has been successful in a proof of concept study of adults with RA (Marsal et al. 2021). Numerous gastroenterology focused editorials have highlighted the importance of the brain-gut axis in the context of IBD and need for neuromodulation based research to determine the therapeutic potential in this arena (Cheng et al. 2020; Bonaz and Bernstein 2013; Bonaz et al. 2017; Browning et al. 2017; Gracie et al. 2019). Accordingly, here we hypothesized that taVNS could be a safe and effective anti-inflammatory therapy in a pediatric and young adult population with CD or UC.

The primary aims of this study were to assess clinical responses and achieving a ≥ 50% reduction in fecal calprotectin (FC) from baseline to week 16. Secondary aims included evaluating the effects of taVNS on various patient reported outcomes (PROs), and heart rate variability as a biomarker of vagal tone.

## Methods

This study was approved by our institutional review board (IRB# 18–0945) before subject enrollment. Subjects were prospectively enrolled between April 1, 2019 and March 1, 2021. Enrollment was paused between March 15, 2020, and August 1, 2020 due to restrictions related to the COVID-19 pandemic. All authors had access to the study data and reviewed and approved the final manuscript.

### Enrollment

**Fig. 1:**
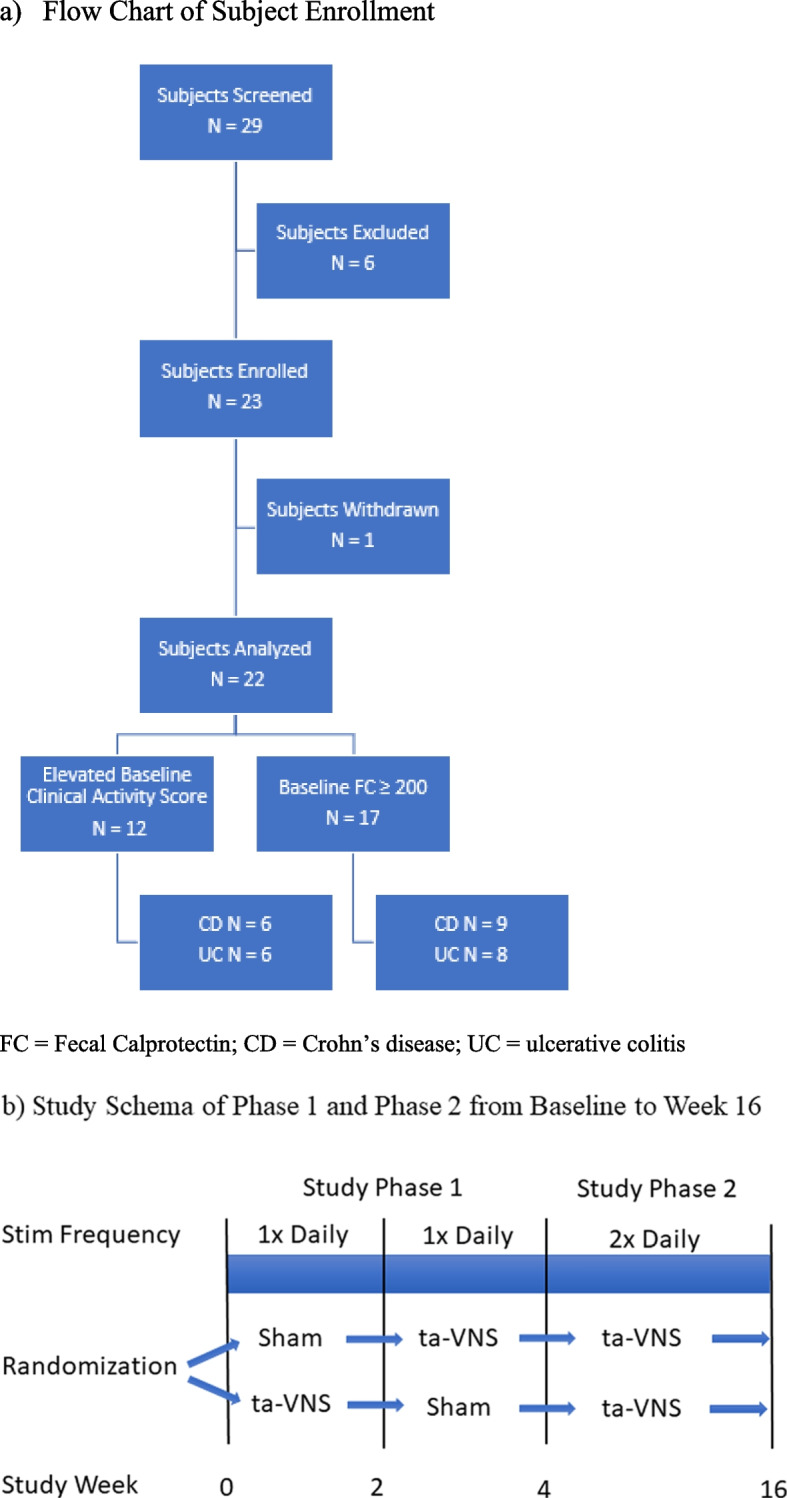
Enrollment and Study Design

Potential subjects between 10–21 years of age with a diagnosis of CD or UC for a minimum of 3 months, and an elevated pre-baseline screening FC ≥ 200 ug/g within 4 weeks of enrollment despite previous treatment with at least one conventional IBD therapy were further screened (Fig. 1a). Subjects needed to be on their existing IBD therapy without dose and interval changes for at least the following amounts of time: oral or rectal 5-aminosalicylate 4 weeks, immunomodulator 8 weeks, and biologic medication 16 weeks. Patients on infliximab were excluded due to potential confounding effects of different infusion intervals on symptoms or calprotectin at time of study visits. Patients were also excluded if they had severe disease activity at the time of screening, described by a weighted Pediatric Crohn Disease Activity Index (wPCDAI) score > 57.5 or Pediatric Ulcerative Colitis Activity Index (PUCAI) score ≥ 65 (Turner et al. 2009, 2017). Additional exclusions included presence of bowel stricture with prestenotic dilatation; intraabdominal or perianal abscess; documented enteric infection in the prior 6 weeks; chronic use of any medication or over-the-counter supplement with cholinergic / anti-cholinergic activity, or nicotine use; known history of arrhythmias or any implanted electronic device; pregnant or lactating females.

### Study design

The study was designed in 2 phases (Fig. 1b). To evaluate the short-term early effect of ta-VNS, in Phase 1 subjects were randomized 1:1 to initially receive either active taVNS (left ear) or sham stimulation of the left calf for the first 2 weeks, performing the stimulation for 5 min once daily. Subjects and their caregivers were blinded to which body location provided active taVNS. Study personnel were not blinded. At week 2, the subject switched to the alternative stimulation site for the next 2 weeks. We were interested in assessing early taVNS responses given disease improvements in 2 weeks or less have been shown in humans with rheumatologic disease (Aranow et al. 2021; Marsal et al. 2021) and with implanted stimulators in preclinical IBD models (Meregnani et al. 2011; Payne et al. 2019).

Phase 2 began at week 4, at which time all subjects were assigned to receive active taVNS via the left ear for 5 min twice daily until week 16. Subjects were instructed to perform one session in the morning and one in the evening, and if not possible then to separate the two stimulation sessions by a minimum of 6 h. Frequency of stimulations were increased to twice daily in this phase to better optimize potential effect of taVNS. All subjects therefore received 14 total weeks of active taVNS. Subjects were assessed at week 0, 2, 4, 8, 12, and 16 with blood and stool samples collected at each visit. Between study visits, the performance of the stimulations was evaluated by a study investigator using virtual telecommunication to ensure accuracy of the stimulation technique and function of the stimulation device. At the conclusion of 16 weeks, subjects were given the option to continue the stimulation.

### Vagus nerve stimulation

**Fig. 2:**
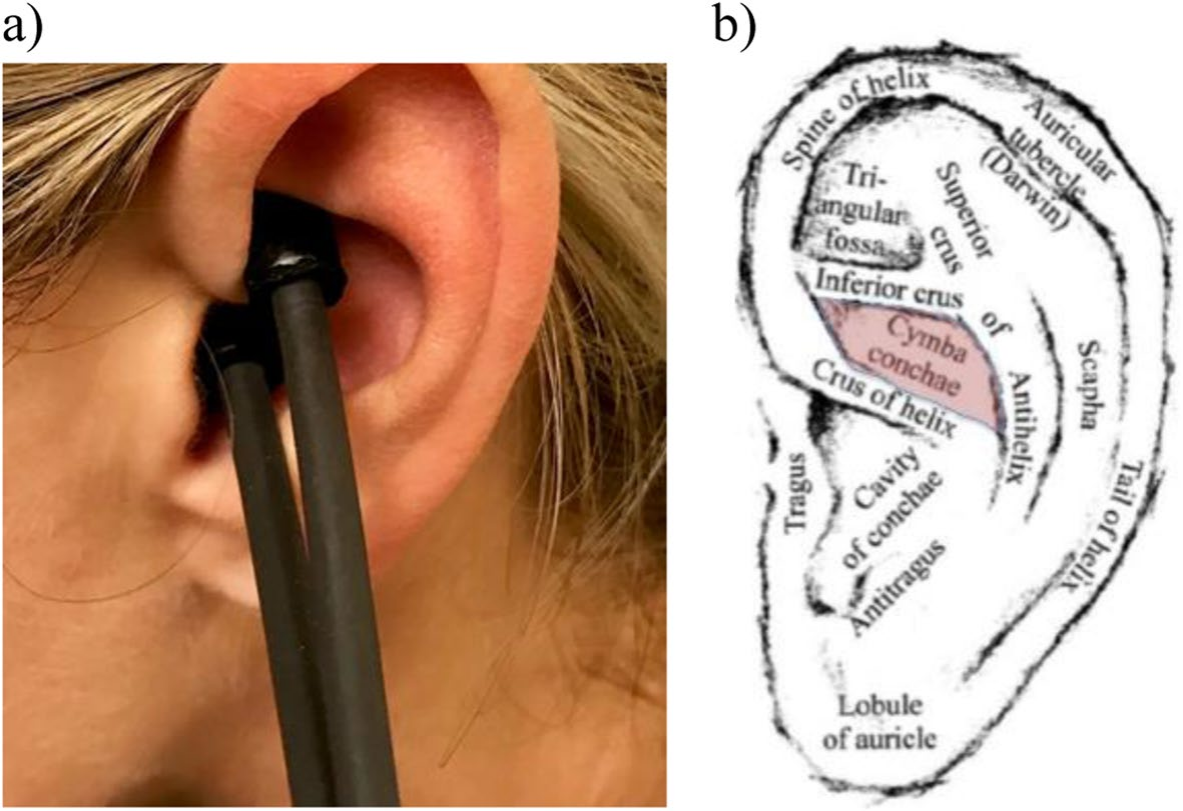
Transcutaneous Auricular Vagus Nerve Stimulation Method. **a** Hand-held sensor probe stimulating the cymba conchae area of the left ear. **b** Schematic of left ear with anatomic location of cymba conchae

A commercial transcutaneous electrical nerve stimulator (TENS) unit (TENS 7000, Roscoe Medical) and a sensor probe (Blue Moon Health) were used to deliver the electrical stimulation (Fig. 2). Conductive electro gel was applied to the tips of the sensor probe. Transcutaneous auricular vagus nerve stimulation was performed at the left ear targeting the cymba conchae, a small area of the external ear between cartilage grooves above the crus of helix (Fig. 2). The TENS unit settings were preprogrammed to deliver continuous asymmetric bi-phasic square pulse waves with a pulse width of 300 µs and frequency of 20 Hz. A timer within the device was pre-set for the desired duration of stimulation, with each stimulation session ending at the completion of the 5-min interval. The intensity of the stimulation was titrated to subject tolerance to achieve the strongest comfortable sensation without eliciting pain. Sham stimulation was performed in the middle of the left calf with the same TENS settings.

### Clinical assessments and patient reported outcomes

Participant weight, vital signs and physical exam were performed at each visit and a wPCDAI or PUCAI score was calculated. For wPCDAI, score ranges of < 12.5, 12.5–40, and 42.5–57.5 represent remission, mild, and moderate disease activity, respectively; whereas PUCAI score ranges of < 10, 10–30, and 35–60 represent remission, mild, and moderate disease activity, respectively. At baseline, week 8, and week 16, a Patient Reported Outcomes Measurement Information System (PROMIS) Pediatric Profile -25 questionnaire (Kappelman et al. 2014) was completed by the study participants. If age < 18 years, a PROMIS Parent Proxy Profile -25 questionnaire was completed as well. PROMIS questionnaires consisted of 4 items in each of 6 domains including physical functioning, anxiety, depression, fatigue, peer relationships, and pain interference. T-score changes > 3 points were considered the minimally important difference to be a significant change.

### Fecal calprotectin

Fresh stool samples were collected within 48 h of each study visit and stored at refrigerated temperature until the study visit, then stored at -20˚ Celsius until the calprotectin assay was performed. FC measurements were performed using the ALPCO Calprotectin Chemiluminescence ELISA assay, with chemiluminescence detection using monoclonal antibodies against calprotectin (ALPCO Diagnostics, Salem, NH, USA). The reportable range for FC in this assay is 7.9 – 6000 µg/g of stool. Samples were run in duplicate to ensure diagnostic accuracy.

### Heart rate variability

Heart rate variability (HRV) was assessed at each study visit, with the baseline assessment conducted before the first transcutaneous stimulation occurred. A continuous 3-lead electrocardiogram was recorded in a standard limb lead II position for 10 min, with the subject in a seated position. HRV was calculated using the SphygmoCor Heart Rate Variability System Software (AtCor Medical Inc, Itasca, IL USA). Vagal tone changes over time were evaluated by HRV power spectrum analysis including low (LF; 0.04–0.15 Hz) and high (HF; 0.15–0.4 Hz) frequency bands and expressed as LF and HF normalized units (LFnu, HFnu), and LF:HF ratio. The LF component represents a combination of sympathetic and parasympathetic nervous system activity while the HF component represents vagus tone (parasympathetic), providing the LF:HF ratio to describe sympathovagal balance; these parameters are modifiable by electrical vagus nerve stimulation (Huston and Tracey 2011).

### Statistics

Summary statistics are given as median and quartiles for continuous outcomes, and frequency and proportion for categorical outcomes. Analysis for fecal calprotectin was restricted to subjects with an FC ≥ 200 at their baseline visit as follows: The association between the change in FC levels at week 2 and at week 4, after 2 weeks of taVNS vs sham stimulation was examined using the exact Mann–Whitney test. Changes in FC levels at baseline vs week 16 were examined using the Wilcoxon signed-rank test. The percent of subjects with a reduction in calprotectin from baseline of ≥ 50% was calculated, along with the associated 95% exact binomial confidence interval. A result was considered statistically significant if *p* < 0.05. As this study was exploratory in nature, no adjustments for multiple comparisons were made. All analyses were conducted using SAS version 9.4 (SAS Institute Inc., Cary, NC).

## Results

### Demographics and patient classification

Twenty-three subjects were enrolled. One subject with prior history of *Clostridioides difficile* infection developed an infection recurrence in the first week after the baseline visit requiring antibiotics and was withdrawn. Baseline characteristics for the remaining 22 subjects are shown in Table 1. Five subjects were between 18–21 years of age at enrollment, and all were diagnosed with IBD prior to age 18. Of note, all 10 of the patients on immunomodulator or biologic medications were on the same medication regimen for at least 6 months prior to study entry. During the study, two subjects with UC were considered taVNS treatment failures for a flare of their disease requiring change in medication; one started prednisone and dropped out after week 12, the other started infliximab after week 12 and chose to continue with taVNS until week 16. Only 3 of 22 had elevated blood C-reactive protein measurements at baseline, precluding further analysis of this inflammatory marker. No one with normal C-reactive protein at baseline had an elevated measurement at week 16.

**Table 1:** Baseline Characteristics

Characteristics	Patients (*n* = 22)
Gender (male) %	12 (55)
Age (years) Median (range)	15 (10–21)
Ethnicity (%)	
Caucasian	14 (63.6)
African American	3 (13.6)
Hispanic	3 (13.6)
Other	2 (9.1)
Baseline Stimulation	
VNS (%)	10 (45.5)
Sham (%)	12 (55.5)
Diagnosis	
Crohn Disease (%)	10 (45.5)
Ulcerative Colitis (%)	12 (55.5)
CD Paris Classification (n)	
L1	3
L2	6
L3	1
UC Paris Classification (n)	
E1	1
E2	1
E3	3
E4	7
Baseline wPCDAI, Median (range)	22.5 (0–57.5)
Baseline PUCAI Median (range)	10 (0–45)
Concomitant IBD Medications (n)	
None	4
5-ASA	8
Methotrexate (monotherapy)	4
Adalimumab^a^	3
Vedolizumab	3

^a^Two patients were on Adalimumab monotherapy. One patient was on combination Adalimumab and Methotrexate

### Phase 1: Baseline to Week 4 Single blinded, sham-controlled crossover

#### Disease activity

Twelve subjects (6 CD, 6 UC) had disease activity scores in the mild to moderately active range at the baseline visit. The median wPCDAI was 27.5 (range 17.5–57.5) for the 6 CD subjects, and the median PUCAI was 25 (range 15–45) for the 6 UC subjects. In the first 2 weeks of active taVNS, 4 subjects (2 CD, 2 UC) demonstrated a clinical response, defined as a reduction in wPCDAI > 12.5 or PUCAI > 10. Two subjects (1 CD, 1 UC) had clinical response while receiving sham stimulation first.

Of the 10 subjects with an inactive wPCDAI or PUCAI at baseline, 3 had increased IBD symptoms while receiving sham stimulation followed by improvement again with taVNS, while 1 receiving taVNS first had increased symptoms at week 2 and reduction in symptoms after sham stimulation at week 4; the remaining subjects had no change in clinical activity in the first 4 weeks.

### Fecal calprotectin

Although all subjects had a FC level ≥ 200 µg/g within the screening 4 weeks prior to baseline, 5 were excluded from further FC analysis for having a baseline FC level < 200. This left 17 of the initial 22 subjects (72.3%) included in the FC analysis. Median baseline FC was 907 µg/g (IQR 411–2,120). In those receiving taVNS first, median FC decreased at week 2, and when therapy was changed to sham, FC sharply increased. Whereas in those receiving sham first, a slight reduction was seen at week 2, followed by a further reduction at week 4 (Table 2). In comparing FC responses between the 2 groups, a statistically significant difference in FC was found between weeks 2 and 4; median Δ FC *increased* by 308 µg/g in those receiving sham and *decreased* by 225 µg/g in those receiving taVNS (*p* = 0.016) (Table 2).

**Table 2:**
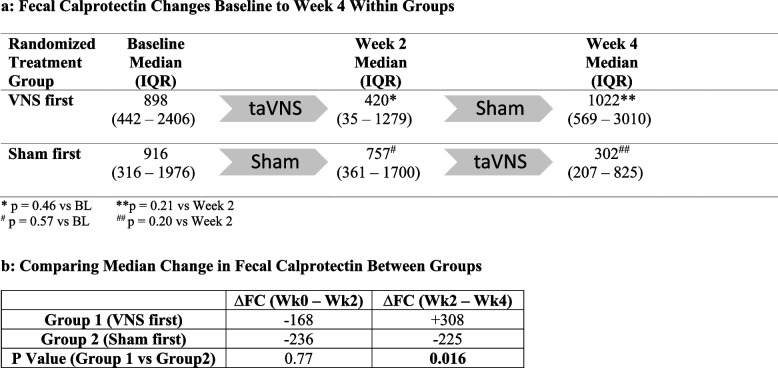
Fecal calprotectin changes in phase I

### Phase 2: Week 4–16 Twice daily active ta-VNS extension

#### Disease Activity

**Fig. 3:**
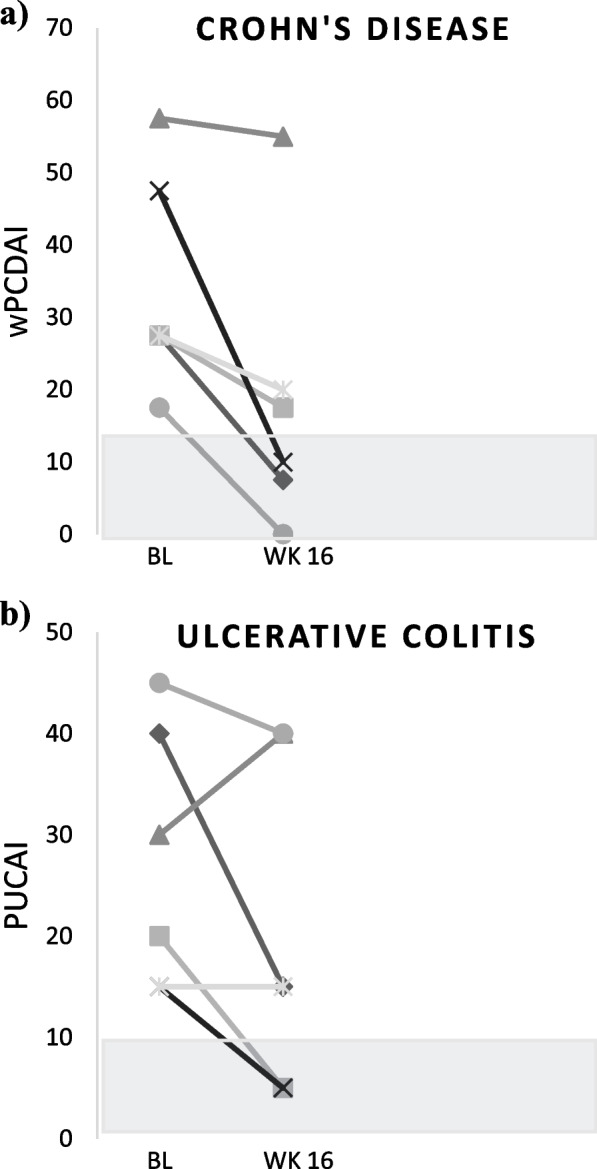
Change in IBD activity index from baseline (BL) to week (WK) 16. **a** wPCDAI = weighted pediatric Crohn’s disease activity index. **b** PUCAI = pediatric ulcerative colitis activity index. Shaded area represents remission zones

At week 16, 3/6 (50%) CD subjects with wPCDAI > 12.5 at baseline achieved clinical remission (wPCDAI < 12.5) (Fig. 3); 2/3 achieving clinical remission also met the primary FC endpoint of ≥ 50% reduction. One subject with CD entered the study with active IBD-associated arthritis, and the arthritis pain resolved after 4 weeks of taVNS stimulation.

Clinical remission (PUCAI < 10) was also achieved in 2/6 (33%) of UC subjects with PUCAI > 10 at baseline, and an additional subject had a clinically significant response with a PUCAI decrease from 40 to 15 by week 16 (Fig. 3); 2/3 of these UC subjects also met the primary FC endpoint.

### Fecal calprotectin

**Fig. 4:**
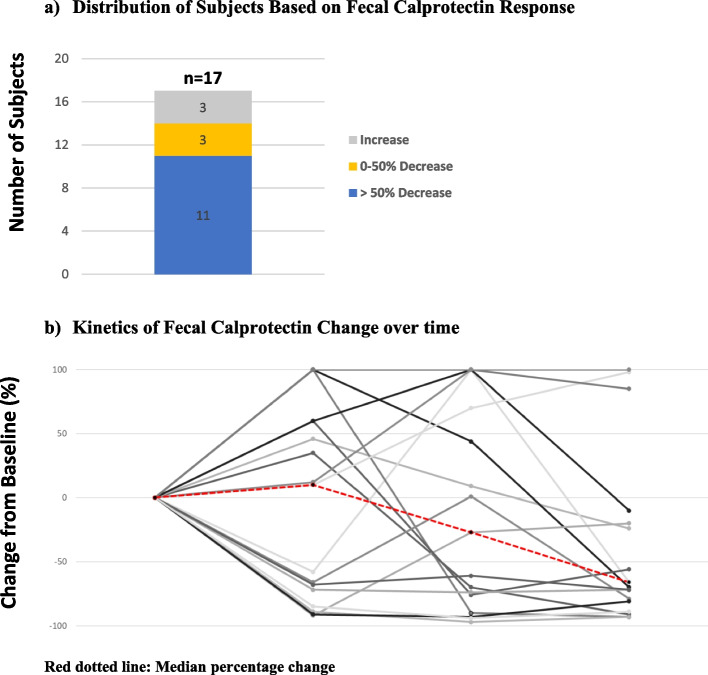
Percentage Change in Fecal Calprotectin from Baseline to Week 16. Red dotted line: Median percentage change

Of the 17 subjects with baseline FC ≥ 200 (9 CD, 8 UC), 11/17 (64.7%) met the primary endpoint of a ≥ 50% reduction in FC at week 16 (95% exact Binomial Confidence Interval: 38.3%-85.8%) (Fig. 4a,b). Seven of the 11 responders had a rapid FC response, with ≥ 50% reduction after only 2 weeks of active ta-VNS, which was maintained to week 16. The remaining four responders did not have any FC improvement during Phase 1, but steadily improved with twice daily stimulation to week 16 (Fig. 4b). At week 16, 4/17 (23%) achieved FC < 100 including 2 subjects (11.7%, 1 CD, 1 UC) who reached normalized FC levels < 50. The subjects who were treatment failures were included as non-responders for calprotectin at week 16.

**Fig. 5:**
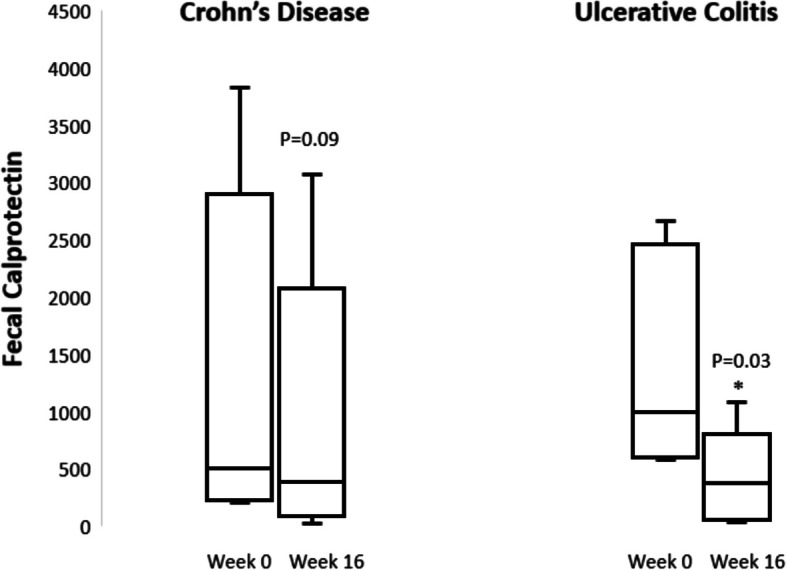
Change in Fecal Calprotectin by Diagnosis

When analyzed by disease subtype, the subjects with CD had a baseline and week 16 median FC value of 506 µg/g (IQR 255–1,976) and 394 µg/g (IQR 149–1,078), respectively, correlating to an absolute median reduction of 357 (IQR -563 to -176) (*p* = 0.09; Fig. 5). The median percent change in FC in the CD cohort was reduction by 56% (*p* = 0.12). Those with UC had a baseline and week 16 median FC value of 994 µg/g (IQR 610–2,265) and 376 µg/g (IQR 83–525), respectively, correlating to an absolute median reduction of 833 (IQR -1,844 to -577) (*p* = 0.03; Fig. 5). The median percent change in FC in the UC cohort was reduction by 81% (*p* = 0.10).

### Patient reported outcomes

Participants and parents completed their respective PROMIS questionnaires at baseline, week 8, and week 16 to assess for any perceived changes in the 6 domains of physical functioning, anxiety, depression, fatigue, peer relationships, and pain interference. T-score changes > 3 points, as the minimally important difference, was noted in the subjects’ anxiety scores and nearly 3 points in the parent proxy anxiety scores. Pediatric median anxiety T-scores decreased (improved) from 46 (IQR 41–55) at baseline to 36 (IQR 36–50) at week 8, and 40 (IQR 36–44) at week 16. Consistent with previous reports, median anxiety T-scores improved in those with clinical symptom improvement and were unchanged in those with stable disease activity. Interestingly however, median anxiety T-scores improved by 6.2 points (Range: -8.2 to -4.1) in the 2 subjects who had *worse* disease activity at week 16. In comparison, parent-proxy median T score improved in the anxiety domain by -2.8. T-score changes in all other domains (physical function, depressive symptoms, fatigue, peer relationships and pain interference) were < 3 points, therefore not significant in differences.

### Heart rate variability

Nineteen of 23 (83%) had complete heart rate variability assessments for analysis. The baseline median LFnu was 57.1 (range 16.4–83.3) and median HFnu was 42.9 (range 16.7–83.6), suggesting lower baseline vagal tone in the full cohort than healthy individuals. Transcutaneous auricular VNS led to an increase in the HFnu (vagal) component of HRV in 4 of 5 subjects with lowest baseline HFnu (< 30), and a decrease in HFnu in all 5 subjects with the highest baseline HFnu values (> 60). The subject with the highest baseline HFnu of 83.6 had a substantial reduction to 29. Modest changes in either direction occurred in the 8/9 subjects with medial HFnu levels (30–60) at baseline; one subject had increase in HFnu from 33.9 to 69.7 over time. This suggests a modulation in vagal tone possibly occurred based on pre-therapy baseline status.

### Patient safety, tolerability, and longer-term outcomes

Transcutaneous auricular VNS was tolerated very well in our patient population. There were no serious adverse events during the study. One subject developed focal redness and a minor break in the skin as result of excessive pressure applied to the skin with the sensor probe in the first week of stimulation. With additional education on the technique of taVNS, this subject did not have any further skin irritation during the study. At conclusion of 16 weeks, 15/22 subjects (68%) subjectively reported improvement in symptoms and chose to continue using taVNS for a period beyond study conclusion. One subject with history of recurrent *C. diff* infection developed an infection recurrence after 3–5 days of taVNS use, which we suspect was unrelated to this therapy given her history and the very short duration of use. Two subjects with UC had worsening disease activity between week 4–12 requiring change in medical therapy; it is uncertain what effect taVNS had, if any, in the progression of disease activity. These two subjects had baseline PUCAI of 30 and 45, respectively, so were already at the higher end of disease activity for the UC subjects enrolled.

Endoscopic assessments were not performed as part of the prospective study. Four subjects continuing to use taVNS as part of their maintenance IBD therapy following conclusion of study participation—without other change in their medication treatments—had a clinically indicated colonoscopy performed by their primary treating gastroenterologist. Medical charts were reviewed retrospectively to compare endoscopic appearance after taVNS use to their most recent colonoscopy before study enrollment. These 4 subject diagnoses, duration of taVNS use until the follow up colonoscopy and mucosal appearance are described in Table 3. The pre-study reference colonoscopy for subject 6, 7, and 9 was performed within 30 days of starting taVNS, while subject 8 had a colonoscopy 9 months prior to starting taVNS.

**Table 3:** Endoscopic Changes Post-taVNS Therapy: Retrospective Review

Study Subject	Diagnosis	Disease location	Pre-study Endoscopic disease severity	Medication at study entry	Duration of taVNS (weeks)	Follow up endoscopic disease severity	Clinical outcome
6	CD	Right colon	SES-CD = 4	None	69	SES-CD = 3	Continued taVNS started UST
7	CD	Left colon	SES-CD = 6	Mesalamine	60	SES-CD = 2	Continued taVNS
8	CD	Left colon	SES-CD = 10	VDZ	16	SES-CD = 4	Continued taVNS
9	UC	Pancolonic	Mayo score 2	Mesalamine	21	Mayo score 2	Discontinued taVNS started VDZ

*SES-CD* Simple Endoscopic Severity of Crohn’s disease

*UST* Ustekinumab

*VDZ* Vedolizumab

## Discussion

In this proof-of-concept clinical trial, noninvasive transcutaneous vagus nerve stimulation delivered via the auricular branch of the vagus nerve resulted in an improvement in clinical symptoms and fecal calprotectin in a pediatric and young adult population with mild to moderate IBD. We demonstrated a ≥ 50% reduction in FC concentration in a significant proportion of our cohort (11/17, 64.7%), which is suggestive of taVNS having an anti-inflammatory effect and possible disease impact in these IBD patients, as no changes in pharmacologic treatment were allowed during the study period or for a period prior to study entry. In the sham-controlled phase of the study assessing early responses, we found 2 weeks of once daily taVNS therapy led to FC reductions, and crossover from taVNS to sham stimulation led to > twofold rise in median FC levels. Between week 2 and 4, a statistically significant change in FC was found comparing those newly on taVNS for 2 weeks to those on sham stimulation (after initially on taVNS the prior 2 weeks). In those who met the FC endpoint at week 16, we found two calprotectin response patterns, with seven responders (2 CD, 5 UC) more quickly improving FC levels at week 4 (2 weeks of active taVNS and 2 weeks of sham), and four responders (3 CD, 1 UC) having no improvement at week 4 and then steadily improving with twice daily taVNS until week 16.

The literature supporting FC as a reliable biomarker of disease activity is robust; reduction in FC concentration has been shown to correlate with endoscopic disease activity, suggest mucosal healing, and predict longer term remission from induction response, (Chen et al. 2021; D'Haens et al. 2012; Molander, et al. 2012) while rising FC has been shown to predict disease relapse (Mao et al. 2012). In subjects from both CD and UC cohorts, a clinically significant FC reduction was seen from baseline to week 16, and a subset achieved normalized calprotectin levels. In the UC cohort, there was a statistically significant decrease in FC levels at week 16, with a median decrease of 833 µg/g. The percent reduction in FC was 81%, and while this change was non-statistically significant (likely due to small sample size), this may be a highly impactful predictor of clinical outcome. A recent report from the PROTECT study, a multicenter inception cohort of children newly diagnosed with UC, found that a FC reduction > 75% from baseline to week 12 of therapy was the best predictor of corticosteroid-free remission at 1 year (Krishnakumar et al. 2022).

Most subjects with baseline disease activity index scores in mild to moderate range had reduced IBD symptoms, including all 6 subjects with Crohn disease. An early 2-week symptom response was not seen to match the early biomarker response. However, importantly, six (3 CD, 3 UC) achieved clinical remission or marked clinical response by week 16. Notably, 4 of these 6 subjects also had a ≥ 50% reduction in fecal calprotectin, indicating symptom resolution could be tied to reduced inflammation as opposed to only effects on visceral pain perception or intestinal motility, which has also been demonstrated (Shi et al. 2021). We were interested in assessing early clinical and biomarker responses to gain insights into the speed of response of this novel therapy. In an adult cohort with Systemic Lupus Erythematosus, taVNS led to symptomatic response within days of starting therapy (Aranow et al. 2021). Further studies isolating CD and UC cohorts is warranted to determine the impact of time to response on remission rates and treatment durability.

Our data are consistent with the anti-inflammatory effect of vagus nerve stimulation seen in prior neuromodulation studies aimed at treating Crohn disease and other immune mediated diseases. In a single center open label trial using a surgically implanted device to treat CD in 9 adult biologic naïve patients, improvements in CRP, FC, and cytokine biomarkers, as well as clinical and endoscopic remissions were found at 12 months follow up (Sinniger et al. 2020). Subsequently, a multicenter trial of adults with biologic-refractory, moderate to severe CD, treated with a surgically implanted vagus nerve stimulator as monotherapy or in combination with biologic therapy was performed. Patients were required to have an elevated FC > 200 ug/g and active endoscopic disease, and at week 16, a majority had reduction in FC concentration and a subset had improvement in endoscopic severity (D'Haens et al. 2019). Vagus nerve stimulation has also been studied in adults with rheumatologic disease. In a study of 18 adults with medication refractory RA, vagus nerve stimulation achieved significant improvement in disease activity scores at day 42 and lowered serum CRP levels (Koopman et al. 2016). Taken together, these results suggest that bioelectronic therapy may be used successfully as a treatment strategy for immune mediated inflammatory diseases.

In addition to improvements in common IBD symptoms and fecal calprotectin, we assessed for benefit of taVNS on health-related quality of life using a validated measure in pediatric IBD patients. We found improved anxiety scores on both the pediatric and parent proxy PROMIS questionnaires, with subjects self-reporting improvements in anxiety at week 8 and sustaining that improvement at week 16. The interplay between anxiety, other mental health outcomes, and IBD is complex, and IBD disease activity is strongly correlated with HRQOL metrics (Brenner et al. 2021). Vagus nerve stimulation is identified as having modulatory effects on the brain gut axis, and has been described as a therapeutic option for neuroinflammation causing various mental health disorders; (Breit et al. 2018) it is therefore reasonable to hypothesize the mental health benefits of taVNS in people with IBD (and other inflammatory disease) could relate to both disease-dependent and independent factors. We were intrigued by the improvement seen in anxiety scores in the 2 subjects with worsening disease activity at study conclusion—albeit a small sample—suggesting further study on this topic is warranted.

We further investigated the effect of taVNS on heart rate variability parameters. Normative HRV values in healthy children have been previously reported, with median HFnu ranging 54–69 depending on calculation method (Gasior et al. 2018). Our IBD cohort had lower baseline HFnu values, suggesting lower vagal tone than healthy peers. Previous studies have found mixed results related to autonomic dysfunction in IBD patients. Prior to regular use of HRV in this assessment, two studies from the 1990s found sympathetic dysfunction in CD and vagal dysfunction in UC (Lindgren et al. 1991, 1993). More recently, low vagal tone has been reported in adult CD patients and associated with high TNFα levels, (Pellissier et al. 2014) and improved HRV metrics has been described in association with less disease exacerbation in pediatric IBD (Yerushalmy-Feler et al. 2022). In our cohort, 4 of 5 subjects with the lowest baseline vagal tone had increased HFnu over time, and all 5 with the highest HFnu values had reduced levels at week 16, suggestive of a recalibration of vagal tone induced by taVNS. A similar pattern has recently been demonstrated in a CD cohort treated with implanted vagus nerve stimulators (Sinniger et al. 2020). A limitation of HRV in this and other studies is the lack of standardized performance and analysis of HRV, including subject positioning and duration of the study, leading analyses to have variability between studies. Further investigation is needed to know if HRV correlates with taVNS treatment response, how children and adults differ in these responses, and how the effects of taVNS compares to pharmacological therapies as disease improves.

The tolerability and safety of taVNS was highly favorable in our study; with children as young as 10 years of age finding this therapy nonpainful and sustainable in addition to the efficacious response many experienced. This favorable safety profile in children was similar to the use of a different neuromodulation treatment for pediatric functional gastrointestinal disorders (Kovacic et al. 2017). Safety is a critical aspect of neuromodulation in the current era of IBD therapy with increasing numbers of biologic and small molecule agents available, and a growing willingness to use these medicines in combination with uncertain longer term risk (Baumgart and Berre 2021; Kwapisz et al. 2021). While current pharmacologic therapies offer essential benefits toward achieving our goals of clinical remission and mucosal healing, there also remains numerous dilemmas in our current treatment paradigms—balancing significant risks with potential benefit, lack of an exit strategy if remission is achieved, managing partial responses without remission, and lack of appropriate therapies for individuals with more mild disease—representing a substantial unmet therapeutic need in patients with IBD. These challenges are all potentially addressed by neuroimmunomodulation (such as vagus nerve stimulation) that could be used as monotherapy or in combination with a conventional pharmaceutical agent. More studies are needed to determine optimal electrical stimulation parameters and understand mechanistic reasons for effectiveness and response variability. Further, future studies with endoscopic endpoints will be needed to confirm mucosal disease modulation and healing. Given the invasive nature of surgically implantable devices (particularly in children), the noninvasive method such as taVNS offers advantages for widespread application in the treatment of chronic inflammatory diseases such as IBD.

Strengths of our study include the prospective study design with early treatment sham comparator, the requirement for elevated fecal calprotectin as inclusion to increase confidence that only patients with active inflammation were being enrolled and controlling for concurrent pharmacologic therapy to better isolate the effect of taVNS on IBD status. Additionally, adherence and accuracy of the stimulation was regularly monitored via telecommunication to confirm the taVNS was performed correctly. Limitations of our study includes lack of a control sham treated group through the full length of the study and lack of endoscopic disease assessments to evaluate the effects of taVNS on mucosal healing. Only subjects were blinded in phase 1 rather than a double-blind assessment, conceivably introducing a bias; study personnel could not be blinded as the sham was a different body part from active taVNS and was known to the investigators demonstrating the therapy. Further, patients were not required to have a symptomatic disease activity index above a particular threshold, leading to a smaller sample size in assessing symptom response.

## Conclusions

Transcutaneous auricular VNS is safe and effective in a cohort of pediatric and young adult patients with mild to moderate IBD, with improvement in symptoms and reductions in fecal calprotectin achieved. Neuromodulation therapies such as vagus nerve stimulation have the potential of becoming an important tool in the treatment toolbox for patients with IBD. Further investigation into noninvasive neuromodulation as an anti-inflammatory therapy with placebo-controlled trials and larger sample size is warranted.

## Data Availability

The datasets generated and analyzed during the current study are not publicly available to protect individual participant information and data but may be available from the corresponding author on reasonable request.

## Abbreviations

IBD: Inflammatory Bowel Disease
CD: Crohn’s disease
UC: Ulcerative colitis
RA: Rheumatoid arthritis
taVNS: Transcutaneous auricular vagus nerve stimulation
FC: Fecal calprotectin
PRO: Patient reported outcome
wPCDAI: Weighted pediatric Crohn’s disease activity index
PUCAI: Pediatric ulcerative colitis activity index
TENS: Transcutaneous electrical nerve stimulator
PROMIS: Patient Reported Outcomes Measurement Information System
HRV: Heart rate variability

## References

[CR1] Addorisio ME, Imperato GH, de Vos AF (2019). Investigational treatment of rheumatoid arthritis with a vibrotactile device applied to the external ear. Bioelectron Med.

[CR2] Aranow C, Atish-Fregoso Y, Lesser M (2021). Transcutaneous auricular vagus nerve stimulation reduces pain and fatigue in patients with systemic lupus erythematosus: a randomised, double-blind, sham-controlled pilot trial. Ann Rheum Dis.

[CR3] Bai A, Guo Y, Lu N (2007). The effect of the cholinergic anti-inflammatory pathway on experimental colitis. Scand J Immunol.

[CR4] Baumgart DC, Le Berre C (2021). Newer Biologic and Small-Molecule Therapies for Inflammatory Bowel Disease. N Engl J Med.

[CR5] Bonaz B, Sinniger V, Pellissier S (2017). Vagus nerve stimulation: a new promising therapeutic tool in inflammatory bowel disease. J Intern Med.

[CR6] Bonaz B, Sinniger V, Hoffmann D (2016). Chronic vagus nerve stimulation in Crohn's disease: a 6-month follow-up pilot study. Neurogastroenterol Motil.

[CR7] Bonaz BL, Bernstein CN (2013). Brain-gut interactions in inflammatory bowel disease. Gastroenterology.

[CR8] Borovikova LV, Ivanova S, Zhang M (2000). Vagus nerve stimulation attenuates the systemic inflammatory response to endotoxin. Nature.

[CR9] Breit S, Kupferberg A, Rogler G, Hasler G (2018). Vagus Nerve as Modulator of the Brain-Gut Axis in Psychiatric and Inflammatory Disorders. Front Psychiatry.

[CR10] Brenner EJ, Long MD, Mann CM (2021). Responsiveness of the Patient-reported Outcomes Measurement Information System (PROMIS) Pediatric Measures to Changes in Disease Status and Quality of Life Among Children and Adolescents With Crohn's Disease. Inflamm Bowel Dis.

[CR11] Brinkman DJ, Ten Hove AS, Vervoordeldonk MJ, Luyer MD, de Jonge WJ. Neuroimmune Interactions in the Gut and Their Significance for Intestinal Immunity. Cells 2019;8(7). 10.3390/cells8070670. 10.3390/cells8070670 PMC6679154 31269754

[CR12] Browning KN, Verheijden S, Boeckxstaens GE (2017). The Vagus Nerve in Appetite Regulation, Mood, and Intestinal Inflammation. Gastroenterology.

[CR13] Chen F, Hu Y, Fan YH, Lv B. Clinical Value of Fecal Calprotectin in Predicting Mucosal Healing in Patients With Ulcerative Colitis. Front Med (Lausanne) 2021;8:679264. 10.3389/fmed.2021.679264. 10.3389/fmed.2021.679264 PMC8369158 34414201

[CR14] Cheng J, Shen H, Chowdhury R, Abdi T, Selaru F, Chen JDZ (2020). Potential of Electrical Neuromodulation for Inflammatory Bowel Disease. Inflamm Bowel Dis.

[CR15] D'Haens GR, Cabrijan Z, Eberhardson M (2019). Vagus Nerve Stimulation Reduced Disease Activity and Modulates Serum and Autonomic Biomarkers in Biologic-Refractory Crohn's Disease. Gastroenterology.

[CR16] D'Haens G, Ferrante M, Vermeire S (2012). Fecal calprotectin is a surrogate marker for endoscopic lesions in inflammatory bowel disease. Inflamm Bowel Dis.

[CR17] Frangos E, Ellrich J, Komisaruk BR (2015). Non-invasive Access to the Vagus Nerve Central Projections via Electrical Stimulation of the External Ear: fMRI Evidence in Humans. Brain Stimul.

[CR18] Gasior JS, Sacha J, Pawlowski M (2018). Normative Values for Heart Rate Variability Parameters in School-Aged Children: Simple Approach Considering Differences in Average Heart Rate. Front Physiol.

[CR19] Ghia JE, Blennerhassett P, El-Sharkawy RT, Collins SM (2007). The protective effect of the vagus nerve in a murine model of chronic relapsing colitis. Am J Physiol Gastrointest Liver Physiol.

[CR20] Ghia JE, Blennerhassett P, Kumar-Ondiveeran H, Verdu EF, Collins SM (2006). The vagus nerve: a tonic inhibitory influence associated with inflammatory bowel disease in a murine model. Gastroenterology.

[CR21] Gracie DJ, Hamlin PJ, Ford AC (2019). The influence of the brain-gut axis in inflammatory bowel disease and possible implications for treatment. Lancet Gastroenterol Hepatol.

[CR22] Hong GS, Zillekens A, Schneiker B, et al. Non-invasive transcutaneous auricular vagus nerve stimulation prevents postoperative ileus and endotoxemia in mice. Neurogastroenterol Motil 2019;31(3):e13501. 10.1111/nmo.13501. 10.1111/nmo.13501 30406957

[CR23] Huston JM, Tracey KJ (2011). The pulse of inflammation: heart rate variability, the cholinergic anti-inflammatory pathway and implications for therapy. J Intern Med.

[CR24] Jin H, Guo J, Liu J (2017). Anti-inflammatory effects and mechanisms of vagal nerve stimulation combined with electroacupuncture in a rodent model of TNBS-induced colitis. Am J Physiol Gastrointest Liver Physiol.

[CR25] Kappelman MD, Long MD, Martin C, et al. Evaluation of the patient-reported outcomes measurement information system in a large cohort of patients with inflammatory bowel diseases. Clin Gastroenterol Hepatol 2014;12(8):1315–23 e2. 10.1016/j.cgh.2013.10.019. 10.1016/j.cgh.2013.10.019 PMC4361943 24183956

[CR26] Koopman FA, Chavan SS, Miljko S (2016). Vagus nerve stimulation inhibits cytokine production and attenuates disease severity in rheumatoid arthritis. Proc Natl Acad Sci USA.

[CR27] Kovacic K, Hainsworth K, Sood M (2017). Neurostimulation for abdominal pain-related functional gastrointestinal disorders in adolescents: a randomised, double-blind, sham-controlled trial. Lancet Gastroenterol Hepatol.

[CR28] Krishnakumar C, Ananthakrishnan AN, Boyle BM (2022). Early Change in Fecal Calprotectin Predicts One-Year Outcome in Children Newly Diagnosed With Ulcerative Colitis. J Pediatr Gastroenterol Nutr.

[CR29] Kwapisz L, Raffals LE, Bruining DH (2021). Combination Biologic Therapy in Inflammatory Bowel Disease: Experience From a Tertiary Care Center. Clin Gastroenterol Hepatol.

[CR30] Lindgren S, Lilja B, Rosen I, Sundkvist G (1991). Disturbed autonomic nerve function in patients with Crohn's disease. Scand J Gastroenterol.

[CR31] Lindgren S, Stewenius J, Sjolund K, Lilja B, Sundkvist G (1993). Autonomic vagal nerve dysfunction in patients with ulcerative colitis. Scand J Gastroenterol.

[CR32] Mao R, Xiao YL, Gao X (2012). Fecal calprotectin in predicting relapse of inflammatory bowel diseases: a meta-analysis of prospective studies. Inflamm Bowel Dis.

[CR33] Marsal S, Corominas H, de Agustin JJ, et al. Non-invasive vagus nerve stimulation for rheumatoid arthritis: a proof-of-concept study. Lancet Rheumatol 2021;3(4):E262-E269. (In English). 10.1016/S2665-9913(20)30425-2. 10.1016/S2665-9913(20)30425-2 38279410

[CR34] Matteoli G, Gomez-Pinilla PJ, Nemethova A (2014). A distinct vagal anti-inflammatory pathway modulates intestinal muscularis resident macrophages independent of the spleen. Gut.

[CR35] Meregnani J, Clarencon D, Vivier M (2011). Anti-inflammatory effect of vagus nerve stimulation in a rat model of inflammatory bowel disease. Auton Neurosci.

[CR36] Meroni E, Stakenborg N, Viola MF, Boeckxstaens GE. Intestinal macrophages and their interaction with the enteric nervous system in health and inflammatory bowel disease. Acta Physiol (Oxf) 2019;225(3):e13163. 10.1111/apha.13163. 10.1111/apha.13163 PMC6519157 29998613

[CR37] Meroni E, Stakenborg N, Gomez-Pinilla PJ, et al. Vagus Nerve Stimulation Promotes Epithelial Proliferation and Controls Colon Monocyte Infiltration During DSS-Induced Colitis. Front Med (Lausanne) 2021;8:694268. 10.3389/fmed.2021.694268. 10.3389/fmed.2021.694268 PMC8292675 34307422

[CR38] Molander P, af Bjorkesten CG, Mustonen H, et al. Fecal calprotectin concentration predicts outcome in inflammatory bowel disease after induction therapy with TNFalpha blocking agents. Inflamm Bowel Dis 2012;18(11):2011–7. 10.1002/ibd.22863. 10.1002/ibd.22863 22223566

[CR39] Ng SC, Shi HY, Hamidi N (2017). Worldwide incidence and prevalence of inflammatory bowel disease in the 21st century: a systematic review of population-based studies. Lancet.

[CR40] Pavlov VA, Chavan SS, Tracey KJ (2018). Molecular and Functional Neuroscience in Immunity. Annu Rev Immunol.

[CR41] Payne SC, Furness JB, Burns O (2019). Anti-inflammatory Effects of Abdominal Vagus Nerve Stimulation on Experimental Intestinal Inflammation. Front Neurosci.

[CR42] Peery AF, Crockett SD, Murphy CC, et al. Burden and Cost of Gastrointestinal, Liver, and Pancreatic Diseases in the United States: Update 2018. Gastroenterology 2019;156(1):254–272 e11. 10.1053/j.gastro.2018.08.063. 10.1053/j.gastro.2018.08.063 PMC6689327 30315778

[CR43] Pellissier S, Dantzer C, Mondillon L, et al. Relationship between vagal tone, cortisol, TNF-alpha, epinephrine and negative affects in Crohn's disease and irritable bowel syndrome. PLoS One 2014;9(9):e105328. 10.1371/journal.pone.0105328. 10.1371/journal.pone.0105328 PMC4160179 25207649

[CR44] Rosas-Ballina M, Olofsson PS, Ochani M (2011). Acetylcholine-synthesizing T cells relay neural signals in a vagus nerve circuit. Science.

[CR45] Shi X, Hu Y, Zhang B, Li W, Chen JD, Liu F. Ameliorating effects and mechanisms of transcutaneous auricular vagal nerve stimulation on abdominal pain and constipation. JCI Insight 2021;6(14). 10.1172/jci.insight.150052. 10.1172/jci.insight.150052 PMC8410029 34138761

[CR46] Sinniger V, Pellissier S, Fauvelle F, et al. A 12-month pilot study outcomes of vagus nerve stimulation in Crohn's disease. Neurogastroenterol Motil 2020;32(10):e13911. 10.1111/nmo.13911. 10.1111/nmo.13911 32515156

[CR47] Teratani T, Mikami Y, Nakamoto N (2020). The liver-brain-gut neural arc maintains the Treg cell niche in the gut. Nature.

[CR48] Tracey KJ (2002). The inflammatory reflex. Nature.

[CR49] Turner D, Hyams J, Markowitz J (2009). Appraisal of the pediatric ulcerative colitis activity index (PUCAI). Inflamm Bowel Dis.

[CR50] Turner D, Levine A, Walters TD (2017). Which PCDAI Version Best Reflects Intestinal Inflammation in Pediatric Crohn Disease?. J Pediatr Gastroenterol Nutr.

[CR51] Windsor JW, Kaplan GG (2019). Evolving Epidemiology of IBD. Curr Gastroenterol Rep.

[CR52] Yerushalmy-Feler A, Cohen S, Lubetzky R, et al. Heart rate variability as a predictor of disease exacerbation in pediatric inflammatory bowel disease. J Psychosom Res 2022;158:110911. 10.1016/j.jpsychores.2022.110911. 10.1016/j.jpsychores.2022.110911 35489164

[CR53] Zhao M, Gonczi L, Lakatos PL, Burisch J (2021). The Burden of Inflammatory Bowel Disease in Europe in 2020. J Crohns Colitis.

